# Computational Approaches in Theranostics: Mining and Predicting Cancer Data

**DOI:** 10.3390/pharmaceutics11030119

**Published:** 2019-03-13

**Authors:** Tânia F. G. G. Cova, Daniel J. Bento, Sandra C. C. Nunes

**Affiliations:** Coimbra Chemistry Centre, Department of Chemistry, Faculty of Sciences and Technology, University of Coimbra, 3004-535 Coimbra, Portugal; djsbento@uc.pt

**Keywords:** cancer, theranostics, nanotherapeutics, imaging, *in silico* models, modeling, simulation

## Abstract

The ability to understand the complexity of cancer-related data has been prompted by the applications of (1) computer and data sciences, including data mining, predictive analytics, machine learning, and artificial intelligence, and (2) advances in imaging technology and probe development. Computational modelling and simulation are systematic and cost-effective tools able to identify important temporal/spatial patterns (and relationships), characterize distinct molecular features of cancer states, and address other relevant aspects, including tumor detection and heterogeneity, progression and metastasis, and drug resistance. These approaches have provided invaluable insights for improving the experimental design of therapeutic delivery systems and for increasing the translational value of the results obtained from early and preclinical studies. The big question is: Could cancer theranostics be determined and controlled *in silico*? This review describes the recent progress in the development of computational models and methods used to facilitate research on the molecular basis of cancer and on the respective diagnosis and optimized treatment, with particular emphasis on the design and optimization of theranostic systems. The current role of computational approaches is providing innovative, incremental, and complementary data-driven solutions for the prediction, simplification, and characterization of cancer and intrinsic mechanisms, and to promote new data-intensive, accurate diagnostics and therapeutics.

## 1. Introduction

The highly intricate nature of cancer makes the approaches to managing and rationalizing large dimensional cancer data notably different from those used in other types of diseases [1,2,3]. Cancer data have been thus associated with myriads of parameters and multiple genome variations and analyzed at the cellular, patient, and population levels [2,4,5,6,7,8], which prevents the establishment of a definite, one-size-fits-all treatment solution.

Although cancer is related to genetic mutations in cells, the interactions between cells and the surrounding medium affect cancer growth and tissue invasion. In order to develop accurate models to describe this highly complex disease, different biological and physiological scales have to be considered and incorporated into mathematical and computational models supporting the rational therapy design. Several approaches have thus provided tailor-made drug treatments towards specific cancer cells, reducing side effects. In this context, different theranostic agents have been developed to selectively deliver the active drug to the tumor site and to simultaneously monitor the therapeutic efficacy by, e.g., constructing tumor imaging frameworks. However, literature regarding cancer theranostics is lacking in comprehensive and systematic approaches to: (1) fully inspect the relevant interaction patterns and synergistic effects, (2) evaluate tumor heterogeneity and data-intensive theranostics technologies, (3) confirm the effectiveness of therapeutics, and (4) compare and validate specific mechanistic models. Fundamental aspects on the cellular and molecular basis of cancer have also been explored through the establishment of relevant biological networks [9,10,11,12,13,14,15,16,17]. This has been facilitated by combining information from cancer genomic, transcriptomic, proteomic, and metabolomic data and computational techniques, aiming at developing non-invasive methods for diagnostic purposes [9]. In addition to several reviews (see e.g., [9,18,19,20,21]), a large number of research papers are focused on the application of metabolomics to specific cancer types, including brain [22], lung [23], prostate [24,25], stomach [26], colorectal [27,28,29], renal [30,31,32], liver [33,34], bladder [35], and oral [36,37] cancer.

*In silico* approaches, including simulation and modelling [38,39,40,41,42,43,44,45,46,47,48], omics [49], and big data [2,48] have supported the tailored design of different therapeutic systems, such as nanoparticles, with optimized properties, providing fundamental knowledge on (1) the molecular basis of the therapeutic system and target cancer, (2) pharmacological performances and on (3) the complex interaction between the designed materials and the target systems [50].

This review provides a timely compilation of the key *in silico* contributions and advances in cancer theranostics technologies. The plenty ways in which computational models and methods are employed to facilitate research of large-dimensional data found in cancer diagnosis, drug development, formulation and optimization, drug repurposing, tumor imaging, and cancer data analytics applications, are also briefly presented.

### 1.1. Connecting Computational Approaches and Theranostics

Establishing the bridge between multivariate cancer data and the ability of models to predict and deal with relevant phenomena, such as drug resistance, tumor heterogeneity and metastasis, and the development of improved therapy procedures, is still a challenge [51].

Mathematical and computational methods have allowed extracting different and complementary data from nanotechnologies, single cell analysis, omics, and big data, among other sources [2,52,53,54]. The main goals of mathematical and computational models developed for dealing with these dynamic and multicomponent systems, displaying multifaceted behaviors, are to reduce research time and cost, suggesting the most profitable strategies for designing in vivo experiments, and producing relevant results to improve patient outcomes, through the theoretical identification of optimal therapies and preventive measures. These models have been tested and compared with preclinical and clinical data, and refined using the available information about the systems under study.

Within the computational strategies, multivariate data analysis techniques and chemometrics, including clustering, unsupervised and supervised dimensionality reduction methods (e.g., principal component analysis (PCA) [9,49,55,56] and partial least-squares (PLS) [49,56], respectively), and non-linear methods such as neural networks (NN) [57] and support vector machine (SVM) [58], are commonly used for achieving fast and reliable results. For instance, while PCA allows for obtaining an overview of the data by summarizing the respective variation into a reduced number of principal components, aiming at building a model for classifying new data samples and identifying target biomarkers, in classification linear methods (e.g., PLS) different biomarkers are readily identified from a model using the loading values [9]. Different statistical methods, including Bayesian estimations and optimization techniques, have been applied to identify unknown model parameters [59,60].

In cancer predictive analytics, different mathematical and machine learning algorithms have also been used to identify the likelihood of future cancer events based on historical data (see e.g., [61,62]). Predictive and descriptive models have allowed, respectively, analysing cancer data and determining the respective behavior based on known attributes and the classification into groups (e.g., genes, cells, tumors, and patients) using descriptive characteristics and historical information. The most widely used predictive models are decision trees [61], regression analysis, and neural networks [9,57,61,62]. Other classifiers include algorithms for clustering, outlier detection, feature selection [61], factor analysis, Bayesian estimations, and SVM [58].

Also relevant is the marked progress of the coupling between imaging technologies (e.g., high content screening, cellular imaging) and computational schemes. The latter have provided deeper knowledge on important aspects of cell and tumor heterogeneity (e.g., relation between genetic and shape heterogeneity) [2,63].

Drug repurposing [64] has also benefited from computational insights, allowing to rationalize and predict important drug–target and drug–cancer relationships by integrating network-, ligand- and structure-based approaches, machine learning, and molecular docking [64].

### 1.2. Relating In Silico and In Vivo Models

**Figure 1:**
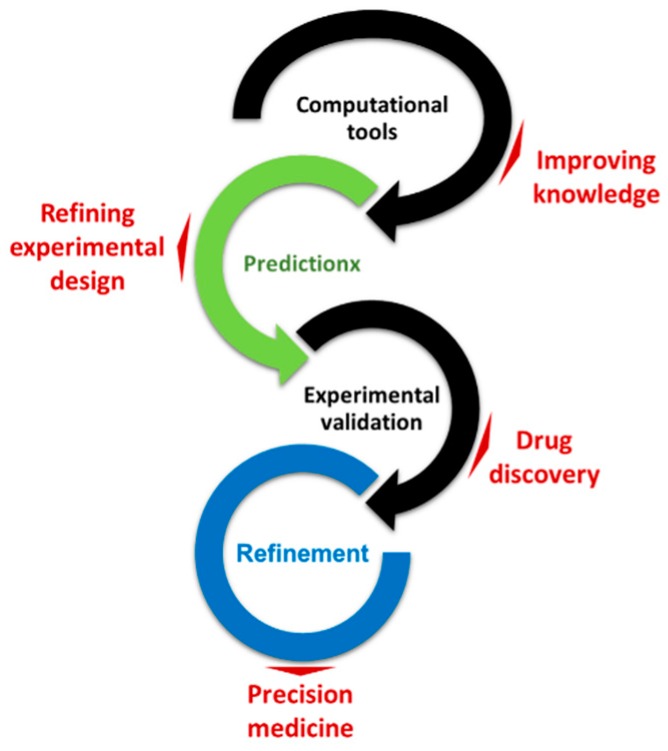
Schematic illustration of the refinement process involving several integrative approaches and powered by new income and technologies. Adapted from [48]. Copyright Taylor & Francis, 2016.

Experimental and computational cancer-based models have provided an invaluable alternative to direct investigations on patients, allowing to overcome experimental design, ethical, economic and welfare issues. However, these models often fail to describe the full complexity of cancer, and in the translational process between preclinical results and clinical scenarios [65]. In spite of this, they have allowed to (1) explain important phenomena associated to intracellular signaling and the respective activation by the pre-selected therapeutic agents, (2) establish a direct connection between intracellular signals and cell behaviors, and (3) simulate the behavior of pathological tissues, predicting the systemic response of the underlying entities [48]. The resulting outcomes can be correlated and validated with in vivo studies and used to improve and fine-tune the models and facilitate predictive-oriented experimental designs (see Figure 1). In turn, data obtained from in vivo studies are used to develop and optimize computational approaches, which are then used as predictive tools to provide new insights on the systems under scrutiny.

Computer models, comprising information on the mechanistic action of drugs, the effects of genetic variants, and the cancer-related signaling pathways, can be applied to drug development and drug-based therapies [48,66]. For instance, the pharmacogenetics of drugs can be determined considering information of the liver. The consequences and side-effects of immunotherapies can be assessed considering relevant elements of the immune system. In addition, individual tumor cells can be modulated to determine a response, reflecting e.g., the heterogeneity of the tumor [67,68,69,70,71]. However, the accuracy of such predictions has been the main hurdle to the regular implementation of computational models. This can be improved using parameter optimization, Monte Carlo, reverse engineering, and model reduction strategies [72,73], as well as artificial intelligence techniques (e.g., deep learning) [67,74]. The benefits and implications of combining computational and preclinical models in drug development and cancer treatment have been duly discussed by Lehrach and co-workers [65]. In general, the model combines information on (1) genes, metabolites, proteins, and relevant cellular/biochemical processes, with an *in silico* description of the cellular signaling network, and (2) alterations of cancer-related molecular entities (e.g., mutated genes and proteins), reflecting the abnormal/reduced activity of oncogenes/tumor suppressor genes [65,68,75,76].

As already mentioned, highly optimized and validated computational models can be developed based on experimental observations, which allow for improving the predictive accuracy of the computational model and also enhancing the preclinical experimental models (e.g., patient-derived xenografting [77,78], genetically engineered mouse models [79,80,81], and organotypic cultures).

**Figure 2:**
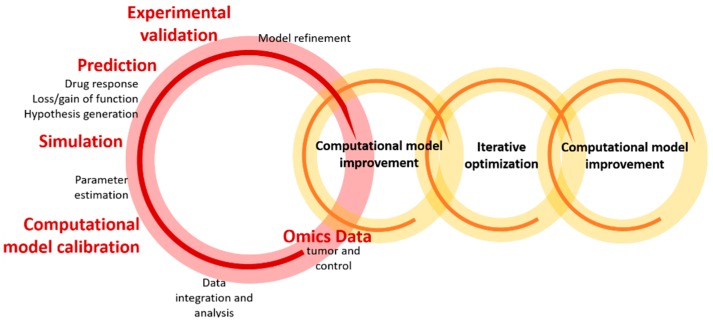
Schematic representation of the iterative optimization workflow, reflecting the data integration/analysis and the development/optimization processes of the computational model. Reprinted from [65] under a CC BY 4.0 License. Copyright Ogilvie, L.A., Kovachev, A., Wierling, C., Lange, B.M.H. and Lehrach, H., 2017.

The optimization and validation of computational models can be conducted imposing different conditions and introducing the parameter and response information in experimental systems using an iterative and flexible scheme, illustrated in Figure 2. The latter enables the identification of relevant parameters, increasing the predictive accuracy of the model and providing the mechanistic details underlying cancer processes and drug action. In simple terms, this iterative procedure involves the integration of omics data (e.g., transcriptome) obtained from experimental samples (e.g., tumor and control samples from patient-derived xenografting, transgenic mice, or organotypic/tumor cell cultures), into a signaling model, which is subsequently trained by these experimental data. The effect of the imposed conditions is simulated using the model parameters, and the expected values are used to validate the resulting predictions in experimental models (see [65,69,82] for details).

The primary goal of collective technologies based on omics data is the ubiquitous detection of genes (genomics), messenger ribonucleic acid, mRNA, (transcriptomics), proteins (proteomics), and metabolites (metabolomics) in a targeted biological sample. The advantages and limitations of these approaches are summarized in [83,84,85,86,87].

The significant progress in genomics and transcriptomics has been fostered by advances in microarray technology. However, the interpretation of microarray data is far from consensual due to the fact that gene expression microarrays quantify alterations in mRNA abundance instead of measuring the protein content. Much information can be extracted from proteomics, which allows understanding the protein function and characterizing internal cell/organ information flow, via protein pathways and networks. This is not, however, suitable for detecting low-content proteins and is hampered by the respective domain size.

Due to the propensity of proteins to be generally affected in cancer and tumor response, the use of proteome data, reflecting genes and the environment, potentiates the discovery of new biomarkers. As the metabolome is the final downstream product of gene transcription and is closest to the phenotype of the system, and the respective alterations are amplified relative to alterations in both the transcriptome and proteome, metabolomics presents advantages over the other omics approaches. Despite the smaller domain of metabolites, metabolome displays higher diversity and chemical complexity, requiring more standardization efforts and accurate and comparative, statistical, predictive, and collecting approaches, for dealing with the intricate nature and heterogeneity of cancer data 84]. The rational and multiscale integration of different types of omics data is essential to elucidate the most relevant factors that lead to the disease and to prioritize treatment targets [88]. Coordinated efforts of consortia displaying complementary expertise and resources are crucial to efficiently translate omics knowledge into the clinic.

## 2. Different Models and Different Scales

A wide range of *in silico* approaches for dealing with information on cancer development, progression, and treatment has been described in an uncorrelated manner in the current state of the art, making the clear distinction and the establishment of relations between them difficult tasks.

In spite of that, a distinction can be made considering the target properties or phenomena to be evaluated at four main scales: the biomolecular, cellular, intercellular/tissue, and macroscopic scales. When the focus is to study the structure and dynamics of proteins, lipids, and other relevant molecules, a biomolecular scale can be considered. At the cellular scale, relevant cell functions, mechanisms, and responses are assessed, while works focusing on cells transformations, such as those occurring in the malignant processes, or in the cell–cell and cell–environment interactions fall into the intercellular/tissue scale. Finally, the tumor morphology, vascularization, shape, and progression are assessed at the macroscopic scale.

In what follows, some selected examples of *in silico* approaches applied to different systems at different scales are presented.

One of the emerging cancer theranostic tools is the use of microRNAs (miRNAs). These small endogenous noncoding RNA molecules regulate gene expression by targeting messenger RNAs (mRNAs), being powerful regulators of the different cellular processes involved in the initiation and development of different types of cancer, including breast [89,90], prostate [91,92,93], thyroid [94], and brain tumors [95].

The fact that miRNAs can be found in biofluids makes them attractive non-invasive tools for cancer detection and monitoring. Moreover, the differential expression of miRNAs has been correlated with cancer development and progression. As miRNAs regulate specific genes, including tumor suppressors and oncogenes, they can be used as therapeutic agents inducing the increase or decrease of the expression level of a given miRNA. When the goal is the inhibition of an oncogenic miRNA, mimics should be used. These are synthetic oligonucleotides identical to endogenous miRNAs able to restore the downregulated activity of miRNA tumor suppressors. Another possibility is the use of miRNA sponges, which are oligonucleotide constructs able to remove the excess of oncogenic miRNAs [91].

Bertoli et al. have explored the role of miRNAs in breast [89,96] and prostate cancers 92,97] employing quantitative analysis. In one of their review papers [97] the authors applied a meta-analysis and a machine-learning approach to their in-depth literature search, for identifying a group of ca. 30 miRNAs with potential for prostate cancer diagnosis and a few miRNAs with prognostic properties. The identified miRNA signatures were validated through a support vector machine model based on a rapid miner workflow.

In two studies [89,96], the authors provided a compilation of miRNAs with capabilities for breast cancer diagnosis, prognosis, and prediction of therapeutic outcomes and also compiled the miRNAs able to increase the efficacy of other non-miRNA treatments. A group of circulating miRNAs that could be helpful in monitoring breast cancer metastasis was also identified [96].

A collection of several computational approaches for identifying cancer-associated miRNAs have been recently reviewed by Cantini and co-workers [98]. The authors found that methods combining miRNA and mRNA expression displayed best performances, with the respective outputs validated in functional experiments. It was suggested that integrating of different data types is the key to improve future development on miRNA-based theranostic tools [98].

The alterations in the information flow over cellular networks, and the structure/function of a protein and the respective interactions with small molecules, are essential for defining and predicting drug response phenotypes, as well as describing the effect of the designed pharmaceutical agents on multiple targets and pathways [48,99].

Some authors [100] have proposed different models aiming at reproducing the behavior of molecules, cells, cancer and drug treatments at the tissue/organ level. Specifically, these models have allowed for (1) describing intracellular and cell–molecule interactions, (2) studying and optimizing organ-specific drug delivery, and (3) predicting the impact of a treatment directly on a given disease outcome or stimulating a response of the immune system. Within the latter, continuous modeling approaches based on differential equations have been commonly employed to model the immune system or its components, allowing to simulate variations in the respective characteristics, actions and concentrations along time, and to identify fundamental aspects of the immune response (see [48,100] for a comprehensive survey of studies evaluating different approaches and models).

One of the most compelling and effective tools for the stoichiometric or dynamic simulation of biological networks [101], and for providing proteome-wide predictions of drug–target interactions and a quantitative modeling of protein–drug interactions, are based on atomistic molecular dynamics (MD) simulation. MD simulations have been performed [102] for exploring the molecular character of BRAF protein, establishing the differences between the wild-type BRAF and mutant BRAF(V600E) protein to unravel the underlying mechanisms of the inhibitor resistance in malignant melanoma. Two potential inhibitors (Aknadicine and 16beta-hydroxy-19svindolinine N-oxide) were elected based on MD, structure-based virtual screening and ligand-based quantitative structure-activity relationship (QSAR) models [48].

The complexity of signaling networks and the respective impact on cellular functions have also been reported. The activity of the epidermal growth factor (EGF) and nerve growth factor (NGF) in rat pheochromocytoma has been simulated by Brown et al. [103]. The EGF model, composed of different protein species involved in several biochemical reactions, allowed for extracting specific and accurate predictions, including the impact of specific signaling modules in the cellular response to the EGF and NGF factors. These results had direct implications in the perception of the effects BRAF alteration on the RAF/ERK and PI3K/AKT pathways [103].

Faura and co-workers [104] developed a logic-based model for predicting intermediate states and identifying relevant topological changes in cell signaling which favor tumor proliferation and drug resistance. Two different frameworks based on this approach have been proposed integrating models that: (1) were constructed based on the prior knowledge of topology and experimental results, and (2) included parameters learned from data using training algorithms [105]. The authors of [105,106] detail some examples of models designed following these frameworks.

Cross-talk among signaling pathways plays an essential role in cancer drug resistance, especially in receptor-targeted therapies, and may occur at multiple levels [107]. However, a systematic understanding on the mechanistic details underlying signaling dynamics mediated by cross-talk and the respective impact on drug resistance, and also information on synergistic drug effects is still missing. We believe that *in silico* methods based on pathway cross-talk inhibition offer invaluable strategies to identifying customized and effective treatments, especially for heterogeneous cancer diseases, such as breast cancer. The use of other oncogenic features, such as gene mutations and the rational identification of biomarkers for pathway addiction, as well as the cross-talk between parallel signaling pathways for identifying patients prone to respond to specific inhibitors, are much needed [107].

Identifying effective drug combinations for treating complex diseases remains a challenge given the large number of possibilities. These have been predicted using different approaches including mathematical modeling, stochastic search, global gene expression, and targeted phosphoproteomics profiling [108]. The mechanisms of action of drug combinations and synergies have been identified resorting to network-based models [15,16,17,108].

The combination of drugs to improving treatment of advanced solid tumors is of paramount importance to bypass drug resistance, promoted by compensatory signaling mechanisms which counterbalance the therapeutic effects and lead to treatment failure [109,110]. The role of pathway cross-talk inhibition in drug resistance has been assessed *in silico* using pathway analysis, which have allowed prioritizing potential drug pairs [107,108,109,110]. Almost 400 anticancer drug combinations have been identified by Aloy and co-workers [108] as potential pathway cross-talk inhibitors, with some of the drug pairs displaying synergistic antitumor effects in human breast cancer cell lines. A drastic potentiation of individual antitumor effects was also found when combining raloxifene, the estrogen response modifier, and cabozantinib, the c-Met/VEGFR2 kinase inhibitor. This represents a compelling framework and a significant advance when comparing the proposed computational network biology approach with combinatorial studies without computational prioritization.

Tumor progression and metastatic dissemination in different cancers have also been the focus of systematic characterization and prediction. Relevant patterns related to dynamic alterations of glioma histological features, radiotherapy efficacy, and survival time have been predicted in cases involving glioma cell proliferation. The velocity of glioblastoma growth and the respective size, as well as the gradient of cells migrating from the border of the tumor, have been determined [100] for monitoring the treatment response. It has been suggested that changes in the kinetics of cells, and also in the proliferation and invasion rates and accumulation of genetic mutations, are not required to reflect malignancy observed in vivo [100].

The correlation between glioma cells and vasculature has also been established based on a model for describing proliferation-invasion-hypoxia-necrosis-angiogenesis. This mathematical model allowed for discriminating histological grades and stereotypical alterations between the tumor grades, and predicting invasion kinetics and malignant scenarios such as hypoxia and necrosis [100].

**Figure 3:**
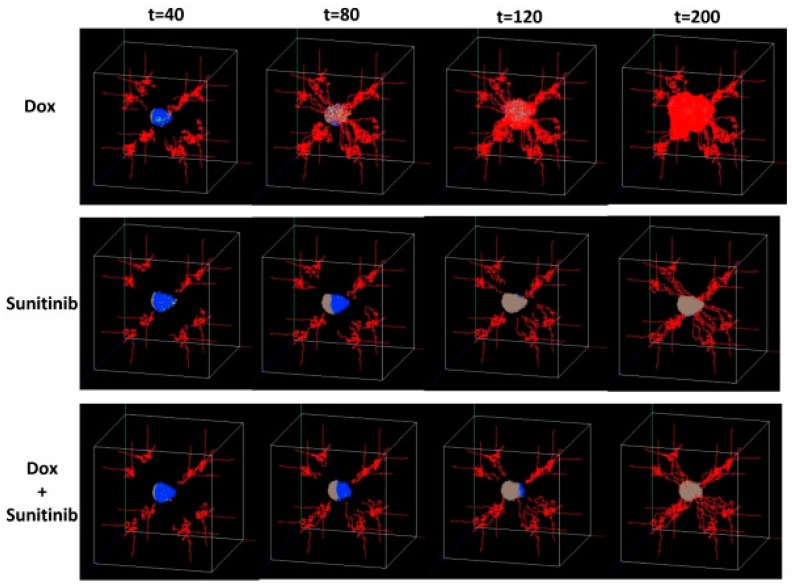
Representative 3D snapshots of the tumor response over time (t = 40, 80, 120, 200 h) and considering three drug-treatment scenarios: doxorubicin alone (Dox), sunitinib alone, and treatment with combined drugs (Dox + sunitinib). Reprinted from [111] under a CC BY 2.0 License. Copyright Wang, J., Zhang, L., Jing, C., Ye, G., Wu, H., Miao, H., Wu, Y. and Zhou, X., 2013.

Zhou et al. [111] designed a multi-scale agent-based model considering the angiogenesis and tumor growth to evaluate the tumor response to a treatment with combined drugs (doxorubicin and sunitinib). The time evolution of the tumor under the treatment with single and combined drugs is presented in Figure 3. For describing this growth process, which has been recognized as a key factor for melanoma’s initiation, progression, and response to treatment, the model integrated the tumor cell and the endothelia cell as two different agents for mimicking, respectively, the tumor progression and vasculature. The multi-scale nature of this model refers to the intra-/intercellular and tissue levels, which describe, respectively, the basic mechanism underlying the phenotypic switch of tumor cells, the connection between tissue and intracellular scales, and the spread of blood vessels through the migration of endothelial cells. It was demonstrated that the role of the tumor angiogenesis interactions in melanoma was successfully described by the model [111]. It was also suggested that the drug-treatment efficacy can be enhanced by interrupting the communications between tumor cells and vasculatures [48].

The fundamental concepts and the critical role of multiscale cancer modeling for providing a quantitative understanding of cancer initiation, progression and treatment, have been introduced and discussed by Deisboeck et al. [112]. This approach refers, essentially, to the simulation of cancer behavior covering multiple biological scales in space and time.

**Figure 4:**
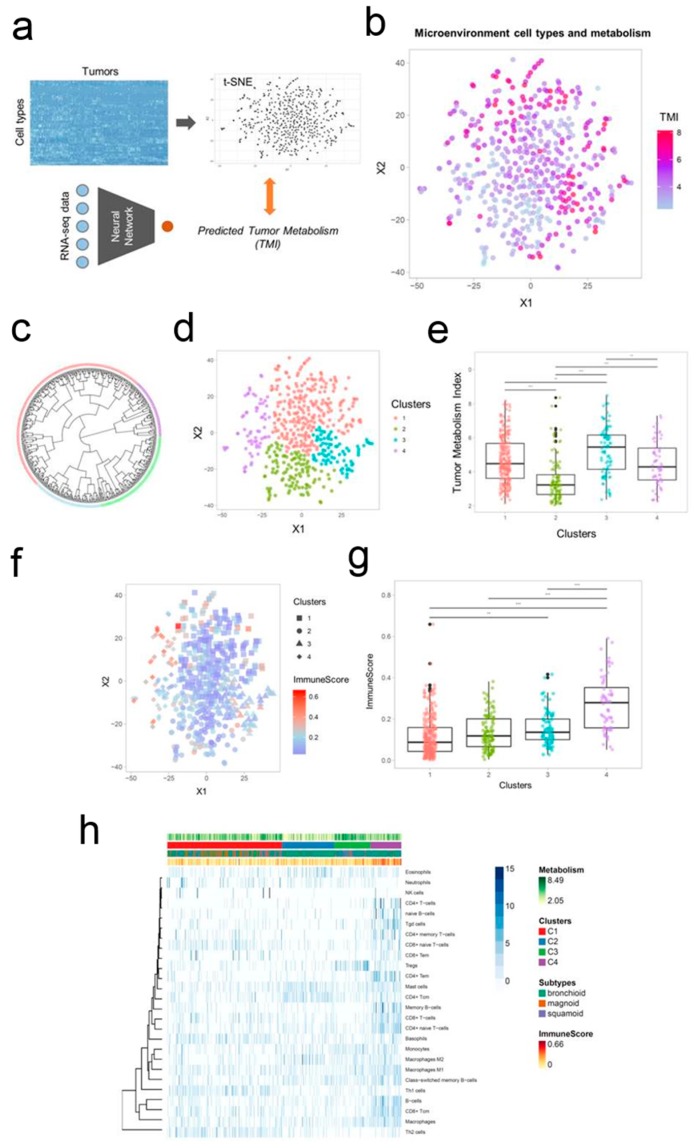
A schematic representation of the two-dimensional projection of cellular landscape of tumor microenvironment. (**a**) Data from The Cancer Genome Atlas (TCGA) were analyzed and represented in a two-dimensional tumor microenvironment landscape map resorting to t-distributed stochastic neighborhood embedding (*t*-SNE) based on the scores reflecting the enrichment of cell types. (**b**) Gradient color representing the tumor metabolism index (TMI) of each sample, showing that hypometabolic samples are placed within populations with low values. (**c**) Circular dendrogram for the groups identified by hierarchical cluster analysis. (**d**) Two-dimensional map of the tumor microenvironment landscape reflecting the similarity between samples. (**e**) Selected clusters displaying significantly different tumor metabolic index (TMI). (**f**) Two-dimensional map of the tumor microenvironment landscape with the ImmuneScore information. (**g**) ImmuneScores were significantly different between the clusters. (**h**) A composed view of the results including the heatmap showing the scores of the enrichment of each immune cell type, the tumor metabolism index, the groups identified based on immune cellular heterogeneity, lung adenocarcinoma subtypes, and ImmuneScore. Reprinted from [113] under a CC BY-NC 4.0 license. Copyright Ivyspring International Publisher, 2018.

More recently, Na and Choi [113] have designed a transcriptome-based tumor metabolism estimation model, using RNA sequencing and image data. This model allowed for estimating cell type enrichment scores and the overall immune cell enrichment. The groups formed based on the cellular heterogeneity in the tumor microenvironment (Figure 4a–e) were further characterized using data from tumor glucose metabolism and immune cell composition (Figure 4f–g).

The four groups identified based on the cellular heterogeneity in the tumor microenvironment (Figure 4c–e) possessed distinct immune cell composition, different tumor metabolism, and a strong correlation with the overall survival. The group possessing high regulatory T cells displayed relative hypermetabolism and poor prognosis and the group with high mast cells and CD4+ central memory T cells displayed relative hypometabolism and favorable prognosis.

A detailed description of the extensive efforts in the field of cancer immunotherapy personalization aiming at integrating clinical data into *in silico* models is provided in ref. [110]. In what follows some selected contributions of modeling approaches encompassing only theoretical models or in combination with clinical information will be presented.

Different studies based on agent-based models [111] and delay differential equations (DDE) [114] have been conducted for exploring combined immunotherapy strategies (e.g., combining activated OT1 cytotoxic T lymphocytes (CTLs) and anti-CD137 monoclonal antibodies) for melanoma treatment in virtual mouse models [66,111]. The agent-based model implemented by Pennisi and co-workers [66] was able to describe the metastatic process and modeling the response of the immune system promoted by a vaccine capable of preventing lung metastases in mice. A reduction of ca. 45% in the number of vaccinations was obtained *in silico*, indicating a potential minimization of side-effects. This approach was inspired in a previous study conducted by Pappalardo et al. [115], focused on the development of in silico experiments for optimal vaccination procedures.

**Figure 5:**
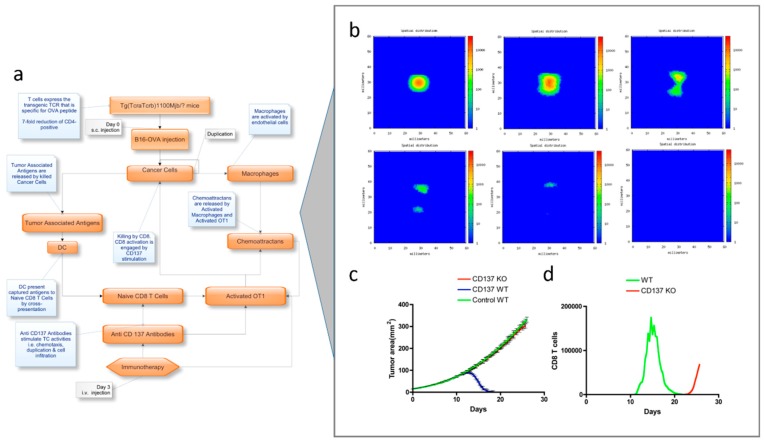
(**a**) Scheme of the modelling workflow, (**b**) Simulation dynamics results of the tumor growth in a virtual mouse after treatment with OT-1 T-cells and anti-CD137 monoclonal antibody, obtained at days 7, 41, 16, 20, 24, and 28 (from upper-left to lower-right). The effect of anti-CD137 in tumor infiltration is confirmed by the increased TC cell cytotoxicity and by the chemotaxis gradients. (**c**,**d**) Model prediction results obtained from the *in silico* study suggesting that the combined therapy with OT-1 T-cells and anti-CD137 monoclonal antibody) was not efficient in eliminating murine B16OVA melanoma with no expression of CD137 on endothelium (**c**). When comparing with wild-type in silico mice, the CD8 T cell infiltration was drastically reduced and delayed in time (**d**). Adapted from [116] under a CC BY 4.0 License. Copyright Pappalardo, F., Forero, I.M., Pennisi, M., Palazon, A., Melero, I. and Motta S., 2011.

Pappalardo et al. [116] have applied this model type to reproduce the tumor progression in a generic tissue section and the effect of an immunotherapy strategy against B16-melanoma. The response of the immune system against tumor cells was evaluated in vaccinated and untreated mice (see Figure 5a). The model allowed for mimicking of specific cell–cell interactions governed by cell receptors using a set of binary strings and reproducing relevant adaptive features, including memory and specificity [116]. Relevant mechanistic details of the tumor progression and of the immune system response against B16-melanoma, as well as the role of CD137 expression on tumor vessel endothelium for achieving a successful therapy, were accurately predicted (Figure 5c,d).

The difficulty in anticipating clinical outcomes according to each patient needs has also fostered the development of innovative modeling approaches capable of analyzing the entire or a specific part of the immunotherapy system, and predicting individual therapy results. These approaches have been formulated based on the integration of patient information with dynamical models representing the impact of different treatment scenarios. Agur and co-workers [117] have recently reviewed these efforts. De Angelis et al. [118] have studied tumor–immune interactions and some competition effects with and without cytokine therapy, resorting to methods supported by generalized kinetic theory, nonequilibrium statistical mechanics and bifurcation diagrams. It was shown that depending on the input parameters, the immune system can be activated and the tumor suppressed, or the latter tends to growth in an uncontrolled manner, prompted by the inhibition of the immune cells. The authors concluded that controlling the inhibition activity of the immune system was decisive for rendering an efficient immunotherapy. Also confirmed was that the excess of activation of the immune system by recytokines, opposing immunosuppression, was useless.

In another study, the cancer-immune dynamics resulting from interleukin-2 (IL-2) or adoptive cell transfer (ACT) immunotherapy was described [119,120], suggesting that a small increase in antigenicity led to significant variations in tumor size. The efficacy of the treatment with single ACT was influenced by the respective intensity and by the tumor antigenicity. The tumor progressed and increased toward a stable stage, in the presence of a reduced number of injected cells and low antigenicity. However, when the amount of cells increased and the antigenicity remained small, two different states are obtained, corresponding to a large or free tumor. This was affected by the initial tumor size, number of cells and concentration of IL-2. The model indicated that single therapy with large doses of IL-2 yielded to the tumor suppression and increased the number of effector cells. The results also revealed that the adverse effects remained using the combined therapy. Other improved strategies have been proposed and are available in [121,122,123,124,125].

For example, the method developed by Cappuccio and co-workers [126,127] for modeling IL-21 antitumor effects revealed that tumor load and IL-21 toxicity were minimized by optimizing the intervals between doses and the dosages, ensuring the highest efficacy/toxicity ratio. The model was calibrated in a following work [128] using data extracted from a preclinical study, involving IL-21 therapy in mice possessing several solid tumors. The accuracy of the predictions were validated using murine-based data acquired from independent experiments and using in vivo experiments encompassing melanoma-induced mice [128].

A similar approach [129] was implemented for investigating the effect of a dendritic cell-based vaccination and the tumor-immune interactions in different treatment regimens. It was observed that the optimal regimen included an initial high-dose and low-dose distributions over the remaining period of treatment.

New insights on prostate cancer immunotherapy have been provided by Agur and co-workers [130] using a personalized model based on differential equations which integrated fundamental interactions between the effects of a vaccine, prostate cancer cells, and the immune system. The model predictions were validated by the results extracted from a clinical trial encompassing an allogeneic prostate cancer whole-cell vaccine. The personalized model was used to predict alterations in the levels of prostate-specific antigen and in tumor burden, emphasizing the feasibility of personalized model-oriented immunotherapy procedures to predict clinical outcomes and improve clinical responses [130]. Also proposed was a solution for optimizing the balance between a proper “in-treatment” validation of the personal model (ensuring maximum model accuracy and minimum realization time), and the primal clinical application of the model-oriented regimen has also been presented [117,131].

An algorithm including information on the preparation, personalization and prediction of a refined treatment, and also on the monitoring stages has been used to validate iteratively personalized models using data from patients, collected as training sets. This algorithm possesses a validation criterion to determine when the personalized model can predict individual treatment results under different treatment procedures [131,132].

Some authors have selected key parameters [133,134] for discriminating treatment responses between patients and explaining the factors underlying treatment failure. For this purpose, grade III was represented by a small value of tumor growth rate and grade IV represented by a larger value, while the other model parameters were fixed at estimated values for the average patient. It was shown that the response patterns observed in the clinical trial (grade III patients with good responses to the alloreactive cytotoxic-T-lymphocytes, in contrast to grade IV patients) [134] can be explained by the aggressive tumor growth of grade IV patients (growth rate is ca. 3-fold higher than grade III).

Other approaches based on the malignant glioma model have been used to explain the treatment failure in slowly growing cancers, characterized by higher initial tumor load or reduced cytotoxic-T-lymphocyte efficacy. With no exception, the desired response can be obtained by increasing the amount of transferred cytotoxic-T-lymphocytes and using the patient’s parameters [117,134].

Very recently, Baker and co-workers [135] have used computer modeling for designing proteins that reproduce the binding sites of cytokines. The proposed strategy allows mimicking the immune-enhancing abilities of interleukin-2 (IL-2), circumventing the intrinsic dangerous side effects associated with the IL-2 dose.

## 3. Optimizing Diagnostic and Therapeutic Agents

It is well known that theranostic approaches refer to systems and strategies in which disease diagnosis and therapy are combined through the administration within a single formulation of a drug and an imaging agent, or a material with an intrinsic ability to be used for imaging purposes, such as gold- and iron oxide-based nanoparticles. The combination of diagnostic and therapeutic agents allows to assess the efficacy of drug targeting or off-target accumulation, to monitor therapeutic efficacy enabling a real-time adjustment of the treatment.

*In silico* approaches can extensively contribute to the development and optimization of these systems, providing a set of numerical modeling methods and computational technologies that support the design of drug and therapeutic systems, allow the prediction of biological interactions and responses, simulate reactions, and estimate optimal parameters [136,137].

Many of the developments in this area have been devoted to the design and optimization of nanotechnology-based systems. The contribution of modeling and simulation techniques is quite vast, relying on the (1) rational design of nanoparticles with optimized characteristics (e.g., shape, size, and surface properties), (2) modelling of drug loading and release profiles, (3) optimization of the internalization processes and interaction with the biological membranes and systems, and (4) improvement of nanoparticles bioactivity, targeting and toxicity, among others [50,137].

Some years ago, Brown et al. [138] found that the coordination of alizarin blue black B to TiO_2_ nanoparticle surface, besides providing a way to discern smaller nanoparticles from the larger aggregates, also enhanced visible light triggered DNA damage upon cancer targets through localized production of reactive oxygen species. Resorting to quantum mechanics calculations at the Density Functional Theory (DFT) level using the B3LYP functional and the polarizable continuum model (IEFPCM) to obtain the free energies of solvation, the authors explored the coordination modes of alizarin blue black B to the TiO_2_ surface, and found that the more favorable interaction occurs through the alizarin blue black B sulfonate group, while the metal center maintained the distorted geometry (see Figure 2 in [138]).

**Figure 6:**
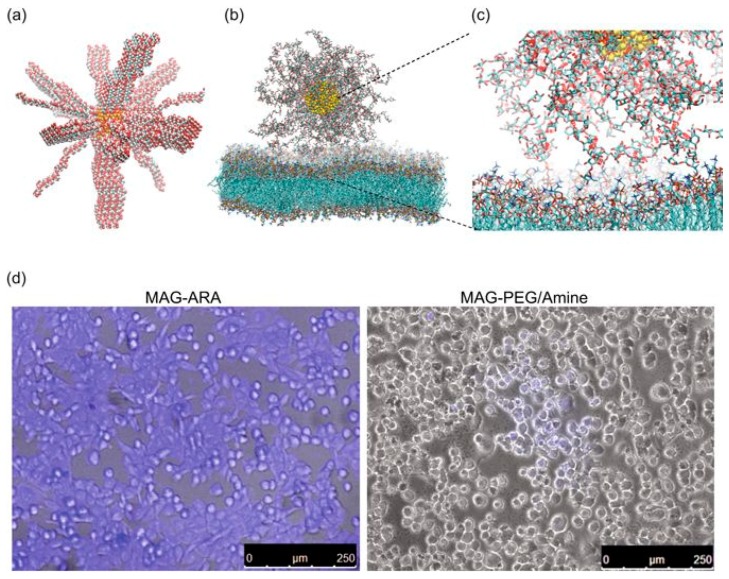
Membrane interaction and internalization of the polyarabic-coated magnetite nanoparticle, MAG-ARA. (**a**) Initial configuration of MAR-ARA in vacuum. (**b**) Final configuration of MAR-ARA in contact with a dipalmitoyl phosphatidylcholine (DPPC) bilayer. (**c**) Example of the interactions of l-arabinose and d-galactose with DPPC. (**d**) Confocal fluorescence image and a bright filed image from human breast cancer cells incubated with MAG-ARA, left, and control nanoparticles, right. Reprinted from [44] under a CC BY 4.0 license. Copyright Patitsa, M., Karathanou, K., Kanaki, Z., Tzioga, L., Pippa, N., Demetzos, C., Verganelakis, D.A., Cournia, Z. and Klinakis, A., 2017.

Recently, Klinakis and co-workers [44] investigated the advantages of coating with polyarabic acid ferrous magnetic nanoparticles to be used as drug carriers and contrasting agents in cancer theranostic. Combining the potentialities of *in silico*, in vitro, and in vivo studies, the authors were able to find out an efficient internalization into breast cancer cells and the excellent drug loading and release behavior of the chemotherapeutic drug doxorubicin (DOX). Using MD simulation the authors explored the interaction between a DPPC bilayer and a magnetite nanoparticle functionalized with a branched polysaccharide consisting of l-arabinose, d-galactose, l-rhamnose, and d-glucuronic acid, with a grafting density of three chains/nm^2^ and chain lengths of 6.5 nm (see Figure 6). The results showed that polyarabic acid interacts with lipid bilayers and efficiently penetrate cells, facilitating the subsequent functionalization through the free carboxyl groups. Furthermore, due to their ferrous magnetic core, the reported system provided excellent in vivo contrasting properties comparable to the commercial contrasting agents used in clinical magnetic resonance imaging.

Also using MD simulation, La Penna and Chelli [45] studied the plasticity of an intrinsically disordered protein involved in tissue remodeling, osteopontin, in order to understand the interactions of the protein in the presence of naturally occurring divalent cations, when it was bound to the respective RNA aptamer and to the theranostic nanoparticle that supported the aptamer.

Fang and co-workers [46] combined MD with binding free-energy calculations to design and select peptides with strong affinity and specificity for positive tumors against human epidermal growth factor receptor 2 (HER2), for imaging and therapeutic applications. These small peptides confer the advantages of small molecule drugs, presenting good membrane permeability and similarly to antibodies were highly target specific and displayed low toxicity.

Minicozzi and colleagues [47] used MD and the standard umbrella sampling procedure to perform binding free-energy calculations towards the design of an engineered peptide, based on the human antimicrobial peptide, LL-37, in order to achieve a more effective binding to the negatively charged model tumoral membranes. Their goal was to propose a methodological strategy applicable in the selection of possible promising carriers directed at theranostics in cancer therapy.

**Figure 7:**
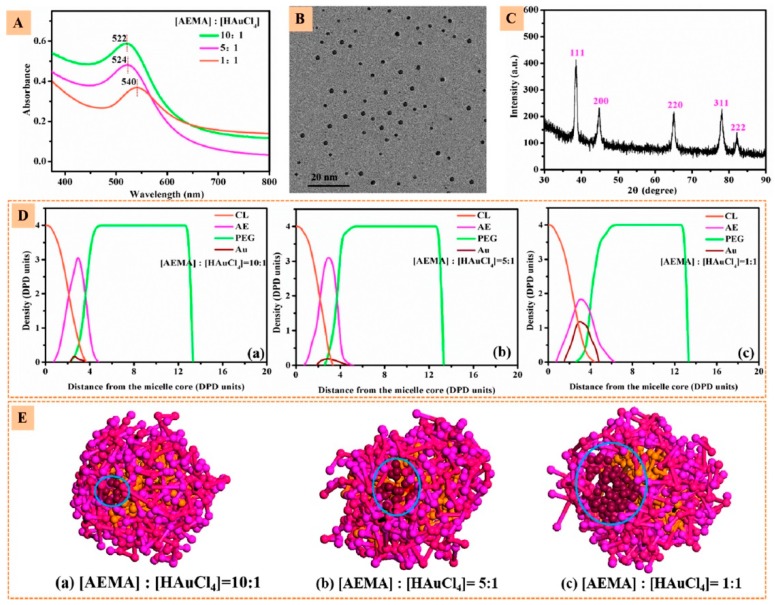
(**A**) UV−vis absorption spectra of β-CD-(PCL-PAEMA-PPEGMA)21/AuNPs at different (AEMA):(HAuCl_4_) molar ratios. (**B**) Transmission Electron Microscopy (TEM) image and (**C**) XRD plot of β-CD-(PCL-PAEMA-PPEGMA)21/AuNPs at (AEMA):(HAuCl_4_) molar ratio of 5. (**D**) Density profiles of different beads and (**E**) cross-section views of β-CD-(PCL-PAEMA-PPEGMA)21/AuNPs at different (AEMA):(HAuCl_4_) molar ratios. Reprinted with permission from [139]. Copyright American Chemical Society, 2017.

Zhang et al. [139] developed a nanovector consisting of a 21-arm star-like triblock polymer of β-cyclodextrin-(poly(ε-caprolactone)-poly(2-aminoethyl methacrylate)-poly(poly(ethylene glycol) methyl ether methacrylate))21 that formed stable unimolecular micelles (β-CD-(PCLPAEMA-PPEGMA)21) that were capable of loading both the computed tomography contrast agent, gold nanoparticles (AuNPs), and the anticancer drug DOX, simultaneously achieving high CT imaging and antitumor efficacies (Figure 7).

**Figure 8:**
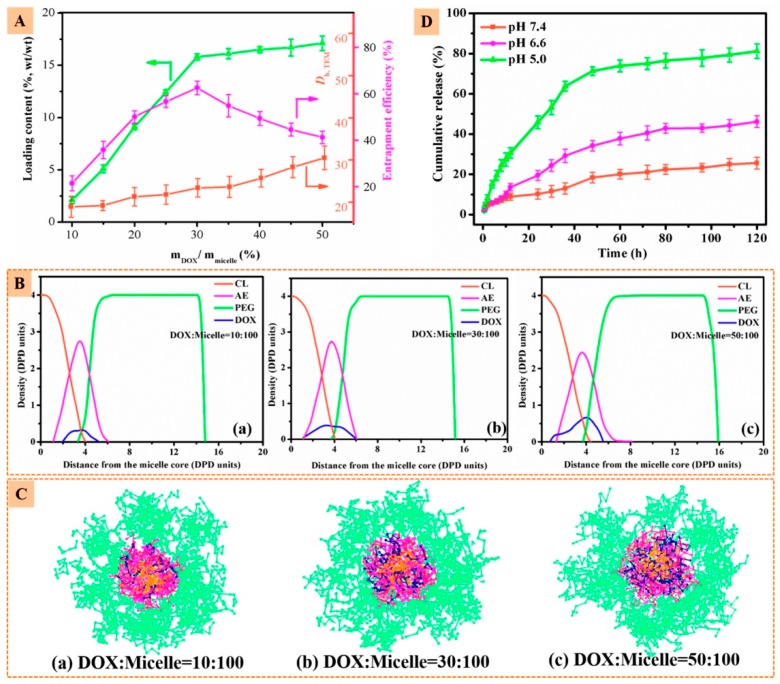
(**A**) Loading content, entrapment efficiency and sizes of β-CD-(PCL-PAEMA-PPEGMA)21/AuNPs/DOX as a function of m_DOX_/m_micelle_. (**B**) Density profiles of different beads and (**C**) cross-section views of β-CD-(PCL-PAEMA-PPEGMA)21/AuNPs/DOX micelles at different weight ratios. (**D**) In vitro drug release profiles of β-CD-(PCL-PAEMA-PPEGMA)21/AuNPs/DOX at different pH levels. Reprinted with permission from [139]. Copyright American Chemical Society, 2017.

Resorting to dissipative particle dynamics simulations with coarse-grained models, the microstructure of the micelles and the preferential location of the nanoparticles and drug molecules on the micelles were enlightened. The nanoparticles and the drug molecule were preferentially located in the PAEMA region, while at higher concentrations DOX penetrated the inner PCL core (see Figure 7 and Figure 8).

The application of nanoparticles to mediated thermal cancer therapy is another issue carefully addressed in [140]. Contributions on this topic range from models in which different shapes of nanomaterials are considered and the effects of nanoparticle heating on biological tissues are explored, to more complex approaches in which cell–nanoparticle interactions, tumor shape, thermal properties of healthy and tumoral tissues, and blood perfusion rates, are incorporated [140].

The patient-specific computational modelling can be a valuable tool to predict tumor proliferation and treatment response. The inclusion of tumor features to elucidate and predict tumor growth and intratumoral drug distribution is a key aspect in theranostics, and its relevance has long been recognized [141,142,143].

Frieboes and co-workers [143] modelled the transport and accumulation of nanoparticles within the tumor microvasculature to monitor tumor growth and drug distribution, considering variations in nanoparticle size and vascular affinity, drug properties, and tumor parameters. They found that for the same vascular affinity small nanoparticles, although with a lower drug loading capability, are more effective than larger ones.

The optimal vascular affinity could be identified providing for proper balance between nanoparticle size, ligand–receptor affinity, ligand density, and the stage of tumor development.

Regarding drug features, the results indicated that increased drug diffusivity is only favorable when nanoparticles are heterogeneously distributed since it enables to achieve a more uniform drug distribution, while it potentiates dispersion in the cases when nanoparticles are already homogeneously distributed.

For many theranostic applications, simulation and image guidance play a crucial role in planning, targeting, and monitoring treatment delivery, being particularly relevant in thermal therapy. Some years ago Fuentes and co-workers [144] explored this strategy and designed a complex structure to remotely handle and visualize the outcome of laser therapy in prostate cancer. Their results showed that simulations based on calibrated nonlinear models of bioheat transfer in heterogeneous tissues coupled with thermal imaging can provide real-time control of the on-going treatment.

Another demand in the optimization of theranostic systems is the effectiveness of the tumor targeting. To meet this goal, Wittrup and Schmidt [145] developed a simple mechanistic model based on empirical relationships to predict the magnitude and specificity of tumor uptake for molecules of different sizes, ranging from small peptides to liposomes. Apart from other features that discriminate these molecules, the size impacts on tumor targeting due to differences in counteracting factors, such as capillary permeability, interstitial diffusivity, available volume fraction, and plasma clearance. Among other interesting results, the authors concluded that tumor uptake as a function of molecule size presented a non-monotonic trend with a minimum uptake occurring for molecules with molecular weight around 25 kDa, with larger and smaller molecules achieving higher tumor levels. Moreover, the uptake of small proteins was fast but required high affinity to be retained while larger molecules with lower affinity achieved higher uptake levels on a much slower time scale. More intriguing was the prediction that tumor uptake of nanoparticles with a dimension around 50 nm was independent of being coupled or not to antigen targeting ligands.

Awojoyogbe and Dada [146] merged the principles of magnetic resonance relaxation to the Bioheat transfer phenomenon, to develop a radiofrequency ablation method to specifically deliver the appropriate amount of heat to the tumor without heating up the surrounding normal tissues. Besides, achieving a significant temperature reduction through the modification of the nature of the applied radiofrequency field, the method also provided real time monitoring of tissues responses during treatment through nuclear magnetic resonance relaxation times and thermal conductivity, which changed according to tissue state. The authors developed a Wolfram Mathematica computer program to assess the tissue thermal responses at varying voxel sizes and exposure times.

In comparison with the current clinical application of hyperthermia, the reported solution, based on a model controlled by relaxation times and biothermal features of the tissues, was able to significantly reduce the time required to attain the desired tissue. The use of radiofrequency pulses confers and the ability to penetrate into deep organs.

## 4. Mapping Multidimensional Cancer Data

Significant advances on computational technologies have improved the ability to accurately diagnose, monitor and control the evolution of cancer diseases.

**Figure 9:**
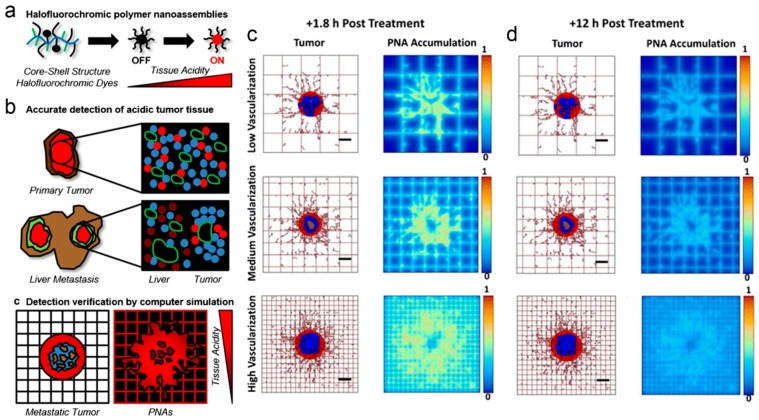
(**a**,**b**) Illustrative description of the strategy used for detecting liver metastatic colorectal tumors using halofluorochromic polymer nanoassemblies (PNAs) and computational simulations. (**c**,**d**) Simulation results reflecting the distribution of the nanoparticles in metastatic tumors at (**c**) 1.8 h and (**d**) 12 h, after systemic injection of the nanoparticles for three different degrees of vascularization. Red refers to viable tumor tissue, blue reflects enclosing hypoxic tissue, and brown reflects necrotic regions. The rectangular grid represents the capillary network, displaying irregular sprouts, which simulate the angiogenesis effect and the growth of blood vessel. Reprinted with permission from [147]. Copyright Springer Nature, 2017.

Bae et al. [147] used a hybrid approach based on computational and experimental methods for designing polymer nanoassemblies bearing halofluorochromic dyes (Figure 9a,b) used for detecting liver colorectal cancer metastasis and improving the treatment response. Results from simulations (Figure 9c,d) suggested that, irrespective of the vascularization degree of the tissue enclosing the lesions, these nanoparticles tended to concentrate in the acidic (hypoxic) interstitium of the metastatic tumors [147].

In acidic microenvironments the nanoparticles displayed a strong fluorescence, which allowed for identifying metastatic tumors. The accumulation of the nanoparticles in hypoxic, acidic regions of metastatic tumors following systemic administration was successfully inspected by computational simulation.

Some authors [148] have also investigated advanced inference methods for mapping data from clinical biomarkers of key biological pathways to reproduce interactions and cooperative effects of signaling proteomic networks in each patient case [149,150]. A new framework [150] for providing case-specific rationales for theranostics has also been proposed.

Other methods to reduce the system complexity and produce relevant visual information for theranostics analysis, including the establishment of hypothesis for precision diagnostics and therapeutics and drug repurposing, have been supported by Resource Description Framework Sketch Maps [148]. The cancer model has been initially defined, reflecting the propagation of the biological signal transduction from the intercellular space via surface proteomic receptors located into the nuclear region in which specific activated protein complexes control gene expression. Start genes including surface receptors, tumor suppressor genes, and end genes corresponding to genes regulating biological processes and involved in carcinogenesis were also defined.

Thanintorn et al. [148] have explored RDF Sketch Maps in hematopoietic cancers (hairy cell leukemia, chronic myeloid leukemia) for which a significant reduction of ca. 20-fold of number of biological entities to be evaluated was obtained, and the most significant entities were retained. For instance, in studies considering pathways related to hairy cell leukemia, it was possible to retain relevant information about signaling cascades leading to the activation of the proto-oncogene BRAF associated with melanoma (see [151,152,153]).

The coupling of biomarker detection and imaging technologies with biological (e.g., pH, hormones) or external triggers (e.g., ultrasound, radiation) for transport of therapeutic agents and to the selected target has been essential for the timely detection, diagnosis, and treatment of cancer.

Biomarkers have been used as input information in artificial neural networks for establishing cancer types and the respective malignancy (see e.g., [10,154,155]). Image fusion techniques have also been used for developing three-dimensional maps of therapeutic doses [156].

Other authors [157] have performed radiation dose calculations for radiopharmaceuticals for improving patient outcomes. In this context, high content screening has for allowed assessing biological/molecular systems using high throughput image phenotyping, providing new insights into relevant alterations in cancer cells (e.g., morphology and shape) and also on different targets for the development of new drugs for specific cancer types (e.g., melanoma) [2].

The used of multi-imaging modalities have revealed important biochemical signatures, the size of the tumors and tracking the respective progression [158]. The use of fluorescent probes, including organic dyes and quantum dots have facilitated the identification and characterization of different internal components of cells and the evaluation of the response to treatment at the cellular level. For example, the most popular imaging modalities incorporating nanoparticles are optical [151] and magnetic resonance [159,160,161,162].

Computational approaches have also contributed to the accurate planning of experiments for delivering increasing doses of radiation therapy to specific target volumes in cancer patients [163,164,165].

Recent advances in magnetic resonance on-line imaging and in the use of implanted markers have allowed enhancing the precise on-time tumor localization, reducing the radiation doses in the surrounding organs.

Molecular imaging has provided relevant information on the tumor phenotype determining treatment decisions and the refinement of the applied therapy by providing predictive data on the treatment outcomes. The delineation of tumor sub-volumes for dose optimization and administration and the identification of spatial patterns of radioresistance are also key applications of molecular imaging [166,167,168].

Different machine learning and artificial intelligence algorithms have provided tools for the automated assessment of tumor response, which have reduced the number of tasks and the variation between readings and increased productivity and efficiency [168]. Advances in radiomics have significantly increased cancer imaging analysis by combining different pattern recognition algorithms (e.g., clustering and feature extraction algorithms), which have facilitated evidence-based clinical decision making. Digital images are transformed into quantitative data that can be combined with genomic, proteomic, clinical and demographic data for determining relevant correlations.

Phenotypic alterations exhibited by human cancers can be observed noninvasively by medical imaging and processed by radiomics, the high-throughput mining of quantitative image features for medical knowledge extraction. Radiomics have allowed improving cost-effective decision support in cancer treatment by (1) converting imaging data into a high-dimensional mineable feature space using several algorithms, and (2) incorporating, diagnostic, prognostic, and predictive signatures. A comprehensive quantification of tumor phenotypes by applying radiomics analysis of a large number of quantitative image features has been proposed by Aerts and co-workers [169]. The selected features were extracted from computed tomography data of patients with lung and head-and-neck cancer and included those features for quantifying tumor image intensity, shape, and texture.

The association of radiomics with clinical factors, prognosis, and gene-expression levels, using large amounts of features and with external/independent validation cohorts of patients, has been evaluated. The authors have shown the translational capability of radiomics in these two cancer types and the successful quantification of a general prognostic cancer phenotype with potential to be employed on other cancer types. The correlation between a prognostic radiomic signature, capturing intratumor heterogeneity and gene expression patterns, was established based on a radiogenomics analysis [169].

Gillies et al. [170] have provided an overview of the challenges, opportunities and implemented tools in the field of radiomics. For instance, analysis of radiomic features derived from magnetic resonance imaging including a Haralick texture analysis have improving the visualization of prostate cancer and the respective intratumoral phenotypic heterogeneity [171].

Different grades of prostate cancer have also been discriminated by radiomics in magnetic resonance imaging [172], contributing to the selection of the most suitable treatment and the implementation of precision strategy.

Also evaluated was the genetic expression classification of the most lethal high-grade serous ovarian cancer. A three-dimensional representation was used for discriminating primary and metastatic lesions. It was found that the longer the distance between a metastatic lesion and the primary tumor, the larger the heterogeneity of the texture between these lesions [173]. It was shown that the reduced overall survival and incomplete surgical resection were potentially explained by the inter-site texture heterogeneity [173].

Xi and co-workers [174] proposed and alternative non-invasive and cost-effective method based on patterns of exhaled aerosols for identifying malignant sites promoted by tumorigenesis in obstructive lung diseases. A combined approach based on computational fluid dynamics and fractal analysis and an image-based lung model, for reflecting the presence and progression of bronchial tumor, was used for evaluating the performance of the method. It was hypothesized that the distribution of exhaled aerosol was specific to the lung structure and sensitive to variations of the airway structure. It was shown that a growing bronchial tumor induced significant variations in the distribution of both airflow and exhaled aerosol, which were more pronounced with the increase in the tumor severity.

**Figure 10:**
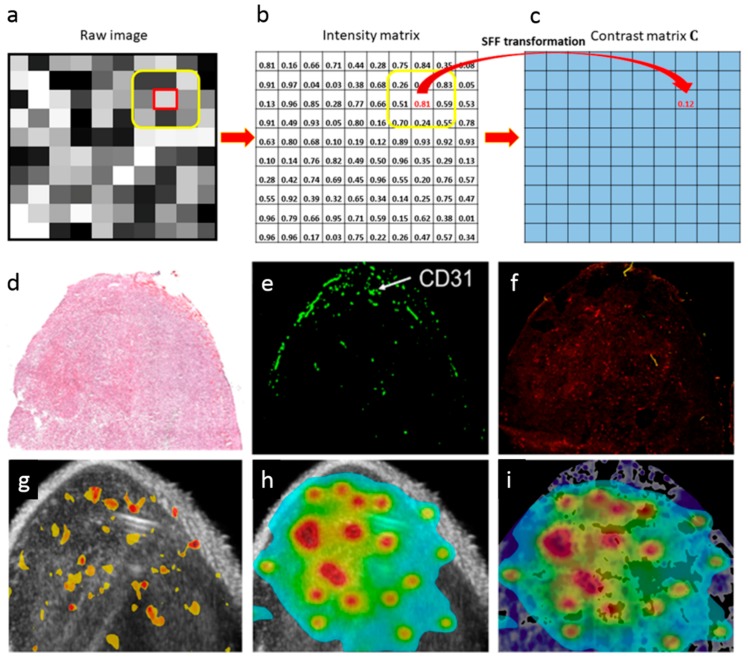
(**a**–**c**) General procedure of the spectral Fiedler field (SFF) method which includes (**a**) a raw image in gray scale, (**b**) the matrix representation of (**a**), and (**c**) the resulting contrast matrix. Using the matrix from of each gray scale image, the method is applied to a small region centered at every single pixel, generating the contrast matrix, which is then processed using a low pass filter for removing noise. The final matrix represents the SFF map. (**d**) Hematoxylin and eosin image of colon tumor. (**e**) Pattern obtained using CD31 endothelial cell marker. (**f**) Fluorescence of the drug in tumors. (**g**) SFF map of echogenic liposomes enhanced ultrasound contrast in tumor areas displaying a positive response for CD31 endothelial cell marker. (**h**) Relative drug diffusion considering the positive regions in SFF map. (**i**) Normalized distribution of doxorubicin combined with doxorubicin fluorescence image. Purple shades refer to doxorubicin fluorescence while blue-red corresponds to the spectral shades of doxorubicin distribution. Reprinted from [175] under a CC BY 4.0 License. Copyright Liu, C., Kapoor, A., VanOsdol, J., Ektate, K., Kong, Z., and Ranjan, A., 2018.

Ranjan et al. [175] developed a spectral Fiedler field (SFF) based-computational method based on graph theory for mapping of nanoparticle in the tumor. This new approach allowed identifying the areas displaying higher liposomal contrast variation in a murine colon cancer model (Figure 10). Specifically, matrix and graph theories were used for assessing differences in surface topology (baseline vs. contrast variations) by transforming contour plots (Figure 10a–c) after nanoparticle injection and measuring over time the dissimilarities between tumor contrast in solid tumors [175].

## 5. Concluding Remarks

The development and improvement of *in silico* models mimicking the mechanistic details of cancer processes is still a fertile ground of research, from the theoretical, empirical and clinical perspectives. The integration of *in silico*/in vitro/in vivo data in preclinical development and the coupling between nanotechnology, molecular imaging, and computer science have offered an invaluable opportunity of attaining effective solutions for the prevention, diagnosis and therapy of cancer. Although there is a general consensus that computational approaches are important tools for the sustained development of accurate and personalized cancer therapies, some concerns still remain pertaining to whether or not those approaches provide reliable support to clinical translation. Comprehensive understanding of cancer-related complications and specificities (e.g., drug resistance, tumor heterogeneity and metastasis), and of tumor genotype, phenotype, metabolomics, and microenvironment, as well as systematic rationales of the involved mechanisms, are still much needed. This means that molecular diagnostics, chemo- and bioinformatics, imaging and big data analytics, and clinical data must be routinely implemented in order to obtain more accurate diagnostic and therapeutic pathways to effectively boost longevity while maintaining the quality of life.
